# Effectively simplified Adriamycin‐induced chronic kidney disease mouse model: Retro‐orbital vein injection versus tail‐vein injection

**DOI:** 10.1002/ame2.12553

**Published:** 2025-01-22

**Authors:** Masaki Watanabe, Hayato R. Takimoto, Kazuki Hashimoto, Yuki Ishii, Nobuya Sasaki

**Affiliations:** ^1^ Laboratory of Laboratory Animal Science and Medicine, School of Veterinary Medicine Kitasato University Towada Japan

**Keywords:** Adriamycin nephropathy, BALB/c, CKD, orbital vein injection, tail‐vein injection

## Abstract

This study aimed to investigate the impact of administration routes in establishing the Adriamycin (ADR)‐induced chronic kidney disease (CKD) model. Using BALB/c mice, we compared the effects of conventional tail‐vein injection (TV10, 10 mg/kg) to those of retro‐orbital sinus (orbital vein) injection (OV10, 10 mg/kg; OV8, 8 mg/kg). The results indicated that the OV10 group exhibited CKD pathology similar to the TV10 group, with both groups demonstrating significantly higher urinary albumin/creatinine ratio (*p* < 0.05), tubular injury (*p* < 0.05), and degree of renal fibrosis (*p* < 0.05) than the OV8 group. No significant differences were observed between the OV10 and TV10 groups in urinary albumin/creatinine ratio, tubular injury, and degree of renal fibrosis. These findings demonstrated that retro‐orbital administration of 10 mg/kg ADR induces comparable effects to conventional tail‐vein administration. This technique's technical simplicity may improve experimental efficiency, reproducibility, and animal welfare in CKD research. In conclusion, this study validates the utility of retro‐orbital injection in CKD model establishment, demonstrating its potential to standardize and improve the reliability of future CKD research protocols.

## Graphical abstract

This study compared the effects of conventional tail‐vein injection and retro‐orbital sinus injection in establishing an Adriamycin (ADR)‐induced chronic kidney disease (CKD) model in BALB/c mice, revealing that 10 mg/kg ADR administered through either route produced similar CKD pathology. The results suggest that retro‐orbital injection is a technically simpler and equally effective method, potentially enhancing experimental efficiency, reproducibility, and animal welfare in CKD research.

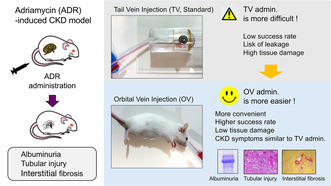

## 1 INTRODUCTION

Chronic kidney disease (CKD), characterized by declining kidney functions and structural changes, affects 8%–16% of the global population.^1^, ^2^ Although diabetes and hypertension are the primary risk factors for CKD, recent studies have identified the role of genetic contributors.^3^, ^4^, ^5^ CKD develops through a complex interplay between environmental, physiological, and genetic factors^1^; this interplay necessitates the development of robust research strategies. In this regard, animal models have emerged as important tools for understanding the underlying mechanisms of CKD. These models allow researchers to conduct controlled studies that may not be feasible in humans, thereby enabling the identification of variables and the elucidation of their effects on kidney functions.

The Adriamycin (ADR)‐induced nephropathy model has been primarily utilized for investigating human primary focal segmental glomerulosclerosis (FSGS).^6^ FSGS is characterized by podocyte injury and loss of glomerular functions, and it commonly leads to kidney damage and failure. This model has been invaluable for exploring the mechanisms underlying FSGS and CKD progression; its advantages include good reproducibility and the ability to induce renal injury with a single dose.^7^, ^8^ Despite these advantages, it has been employed by relatively few researchers, presumably because of the technical difficulty in administering lateral tail‐vein injections of ADR. This route of administration is particularly challenging due to difficulties in visualizing and accessing small blood vessels, which requires precise insertion techniques and proper animal restraint. Additionally, the technique induces significant stress on the animal subjects, potentially affecting experimental results.^9^

Comparative studies involving the administration of small molecules (e.g., radiodiagnostics and radiopharmaceuticals) have demonstrated that the retro‐orbital administration route is just as effective as the tail‐vein route.^9^ Retro‐orbital injections have been routinely used for bone marrow transplants, leukemia induction, administration of experimental compounds, and gene therapy.^10^, ^11^ Importantly, by analyzing postmortem tissue samples, it has been demonstrated that the technique does not damage the morphologies of the sinus and surrounding tissues.^12^ Thus, this technique is generally considered a simple and reliable method for intravenous administration of various compounds.

However, the utility of retro‐orbital administration for the ADR‐induced nephropathy model has not yet been investigated. The aim of this study was to examine the differences in CKD pathology between the tail‐vein and retro‐orbital administration routes for intravenous injection of ADR in BALB/c mice. Ultimately, the optimal dosage of ADR for retro‐orbital administration was determined to establish a new, technically simpler model of ADR‐induced nephropathy.

## 2 MATERIALS AND METHODS

### 2.1 Animals

BALB/c mice are commonly used to establish ADR‐induced nephropathy models due to their high sensitivity to ADR‐induced kidney damage.^13^ We utilized these mice in this study to compare our results to the previously reported results.^7^, ^8^ BALB/cAJcl mice were purchased from Clea Japan (Tokyo, Japan) and acclimatized for 2 weeks at 22 ± 2°C and 40%–60% relative humidity under a 12‐h light/dark cycle. The mice were provided with standard laboratory diet, CE‐2 (Clea Japan), and tap water.

### 2.2 Study design

In this study, we evaluated the effects of administration route and dosage on an established ADR‐induced CKD model in an 8‐week‐old male BALB/c mice (*n* = 30). Mice were randomly divided into three groups (*n* = 10/group) and received a single dose of ADR as follows: retro‐orbital injection of 10 mg/kg (OV10), retro‐orbital injection of 8 mg/kg (OV8), and tail‐vein injection of 10 mg/kg (TV10; control group). According to previous studies, we set 10 mg/kg as the standard dose of ADR for this model.^6^, ^7^

### 2.3 Administration route of ADR

For the tail‐vein injections of ADR (doxorubicin hydrochloride; Fujifilm Wako Pure Chemical Corp., Osaka, Japan), at a dose of 10 mg/kg, the standard protocol was utilized.^7^ The mouse was first secured in a restrainer (item number 01318, SANPLATEC Corp., Osaka, Japan), and its tail was rubbed with an alcohol swab to disinfect and dilate the vein. Using a 29‐gauge needle, the drug solution was injected into the tail vein at a shallow angle, ensuring a slow and careful injection to avoid damage. After the injection, the needle was carefully removed, and pressure was applied with a finger to stop any bleeding. For the retro‐orbital injections of ADR, a previously described method was utilized.^12^ The mouse was first anesthetized in an inhalation chamber supplied with isoflurane. After anesthesia, the mouse was positioned in left lateral recumbency with its right eye facing upward. The eyeball was gently pressed, and a 29‐gauge needle was used to inject ADR at a dose of 8 or 10 mg/kg to the inner canthus. The needle angle was set at approximately 30 degrees, and the needle tip was inserted along the lower edge of the eyeball. The solution was slowly and smoothly injected. After the injection, the needle was slowly withdrawn.

### 2.4 Urinary albumin level

Urinary albumin (Alb) was collected from the bladder of the mouse subjects 4 weeks after ADR administration and detected using sodium dodecyl sulfate‐polyacrylamide gel electrophoresis (SDS‐PAGE). As the positive control, 5 μg of bovine serum Alb was loaded simultaneously. After electrophoresis, the gel was stained with Coomassie Brilliant Blue (CBB) using a Rapid Stain CBB kit (Nacalai Tesque, Kyoto, Japan) and destained in distilled water (DW) for 24 h. The staining image was captured using a commercial scanner (GTX‐820, EPSON, Nagano, Japan). The CBB‐stained band corresponding to Alb was quantified using the ImageJ software (https://imagej.nih.gov/ij/, National Institutes of Health, Bethesda, MD, USA). In addition, urinary creatinine (Cre) levels were measured from the same urine samples using a Creatinine Colorimetric Assay kit (Cayman Chemical, MI, USA), following the manufacturer's instructions. The measured Alb levels were corrected for the urinary Cre levels, and the Alb/Cre ratio was calculated.

### 2.5 Histology

Harvested kidneys were fixed in 4% paraformaldehyde and embedded in paraffin. Sections were prepared in thicknesses of 2 and 4 μm from the paraffin blocks for periodic acid–Schiff (PAS) and picrosirius red (PSR) staining, respectively. According to established protocols,^14^ sections were treated with 0.5% periodic acid solution for 5 min, washed in distilled water, stained with Schiff's reagent (Fujifilm Wako Pure Chemical Corporation) for 30 min, washed thrice with 0.8% sodium bisulfite solution for 3 min each, rinsed in running water, counterstained with hematoxylin for 4 min, washed in running water, rinsed in distilled water, cleared, and mounted. Kidneys were used for PSR staining 28 days after ADR administration. Based on previous studies,^14^ tissue processing and staining were conducted as follows: the sections were subjected to deparaffinization followed by washing in running water and a brief rinse in distilled water for 60 s. They were then stained for 1 h using solution A, composed of 0.5‐g sirius red dissolved in 500 mL of saturated picric acid solution. Subsequently, the sections were treated thrice with solution B (5 mL of acetic acid in 1000 mL of distilled water) for 3 s each. Finally, the sections were cleared and mounted.

Tubule heights in the renal cortex were measured using the Image J software. These measurements were taken in three randomly chosen fields at 100× magnification. The average value per 50 tubules was calculated. Fibrotic area quantification was performed by taking three randomly selected fields at 40× magnification in the renal cortex, and the stained areas were measured using the Image J software to calculate the percentage of renal section area. Glomeruli and vessels stained with PSR were excluded from the measurement used to assess tubulointerstitial fibrosis. The experiments were conducted by one individual, whereas the data analysis was performed by another person in a blinded manner.

### 2.6 Statistics

All data are presented as mean ± standard deviation (SD). One‐way analysis of variance (ANOVA) was used to statistically analyze parametric data among multiple groups, followed by the Tukey–Kramer post‐hoc test for pairwise comparisons. Survival curves were generated using the Kaplan–Meier method, and the log‐rank test was used for testing. A *p*‐value < 0.05 was considered statistically significant. All statistical analyses were performed using JMP Pro 17 (SAS Institute, Cary, NC, USA).

## 3 RESULTS

### 3.1 Urinary Alb excretion

**FIGURE 1:**
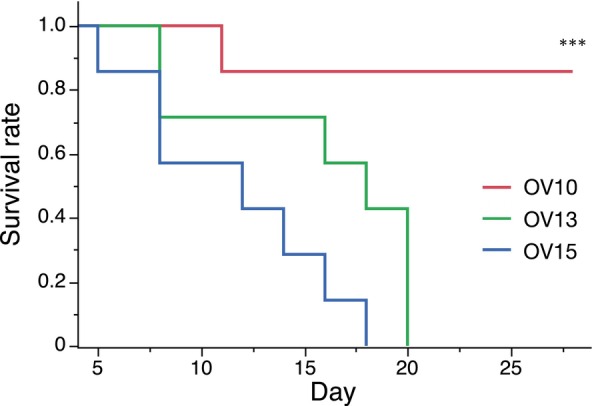
Log‐rank survival analysis of administered with retro‐orbital injection mouse. Survive rates of retro‐orbital injection of 10 mg/kg Adriamycin (ADR; OV10, *n* = 7), retro‐orbital injection of 13 mg/kg ADR (OV13, *n* = 7), and retro‐orbital injection of 15 mg/kg ADR (OV15, *n* = 7) were analyzed. ***p < 0.001.

For a pilot study, ADR was administered via the retro‐orbital route at doses of 15, 13, 10, and 8 mg/kg (*n* = 7/group). It was observed that in the groups receiving 15 and 13 mg/kg doses, all mice had died by 28 days postinjection, indicating severe pathology incompatible with chronic conditions (Figure 1). Therefore, we compared the groups receiving 10 and 8 mg/kg doses administered via the retro‐orbital route to the group receiving a 10 mg/kg dose administered via the tail‐vein route.

**FIGURE 2:**
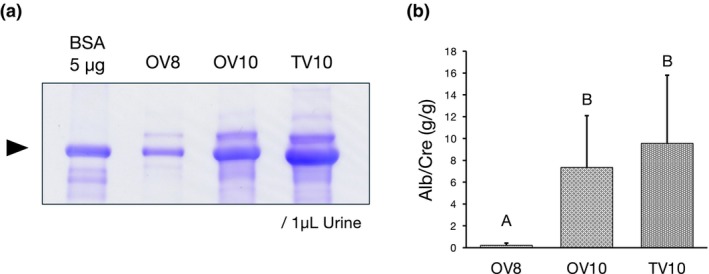
Urinary albumin excretion in mice treated with Adriamycin (ADR) via different administration routes. (a) Sodium dodecyl sulfate‐polyacrylamide gel electrophoresis (SDS‐PAGE) image of representative individuals administered with retro‐orbital injection of 8 mg/kg ADR (OV8), retro‐orbital injection of 10 mg/kg ADR (OV10), and tail‐vein injection of 10 mg/kg ADR (TV10). Bovine serum albumin: BSA. (b) Urinary albumin excretion levels of OV8 (*n* = 10), OV10 (*n* = 10), and TV10 (*n* = 10), 28 days after ADR administration. A versus B: *P* < 0.05.

In CKD, the progression to end‐stage renal failure involves an initial phase with disease‐specific pathology, which is often followed by a common clinical course of glomerular injury leading to proteinuria, tubular damage, and interstitial fibrosis.^15^, ^16^ Thus, we first assessed the proteinuria levels in the OV10, OV8, and TV10 groups. The Alb/Cre ratios of OV10 and TV10 were significantly higher than that of OV8, with no significant difference between OV10 and TV10 (Figure 2A,B).

### 3.2 Histological investigation

**FIGURE 3:**
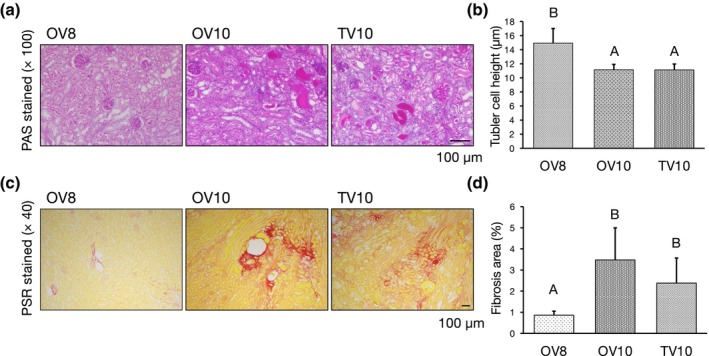
Histological analysis of kidneys harvested from Adriamycin (ADR)‐treated mice. (a) Representative periodic acid–Schiff (PAS)‐stained renal sections. (b) Tubular heights of OV8 (*n* = 10), OV10 (*n* = 10), and TV10 (*n* = 10) were analyzed. A versus B: *P* < 0.05. (c) Representative picrosirius red (PSR)‐stained renal sections. (d) Fibrosis area versus total area of OV8 (*n* = 10), OV10 (*n* = 10), and TV10 (*n* = 10) was analyzed. A versus B: *P* < 0.05.

Tubular injury and tubulointerstitial fibrosis are common pathological features of CKD that contribute significantly to decreased renal functions.^17^ Tubular injury is characterized by tubular dilation, flattening of tubular epithelial cells, loss of brush border, thickening of the tubular basement membrane, detachment of tubular epithelial cells, and intracellular vacuolization. Tubular atrophy is a hallmark of CKD that indicates the extent of tubular damage, which can be quantitatively assessed by measuring the height of proximal tubules.^18^ This measurement serves as a valuable marker for evaluating the severity of tubular injury. The tubular heights of OV10 and TV10 were significantly lower than that of OV8, with no significant difference between OV10 and TV10 (Figure 3A,B).

Fibrosis is another common pathological feature of CKD, in which damage to the renal tissue leads to scar formation.^19^ The degrees of fibrosis (% of fibrotic area) of OV10 and TV10 were significantly higher than that of OV8, with no significant difference between OV10 and TV10 (Figure 3C,D).

## 4 DISCUSSION

In this study, we examined how the method of administering ADR, either through the tail‐vein or through the retro‐orbital sinus, affects the development of CKD. Additionally, we identified the optimal dosage of ADR for retro‐orbital administration to establish a technically manageable approach for this disease model. We demonstrated that retro‐orbital administration of ADR (10 mg/kg) produced similar results to the conventional tail‐vein injection method in terms of CKD development. This finding has significant implications for improving experimental protocols in CKD research.

Mouse tail‐vein injection is a commonly used technique in mouse studies, but it poses several challenges. It requires considerable skill and practice to perform accurately due to the small size of tail vein in mice.^12^ These technical difficulties can make the animal procedure extremely time‐consuming, especially when handling a large number of mice. Furthermore, improper administration can lead to complications such as local tissue damage, hematomas, or leakage of the injected substance, which can impact research outcomes.^11^ Because this procedure is performed without anesthesia, it often causes stress on the mouse subjects. This stress can be exacerbated when multiple attempts are needed for successful administration. Collectively, these factors can introduce variability in experimental results, potentially affecting the reliability and reproducibility of the obtained data.^11^

Retro‐orbital injection is technically simpler and faster than tail‐vein injection.^12^ It is performed under anesthesia and does not require prolonged restraint needed for tail‐vein injection, thereby significantly minimizing stress on the mouse subjects. In this study, OV10 demonstrated similar effects to TV10 in terms of proteinuria levels and histological changes. This suggests that retro‐orbital administration can serve as an effective alternative to the conventional tail‐vein administration.

Furthermore, retro‐orbital administration offers several technical advantages over tail‐vein administration. It can potentially improve the visibility of the injection site and enhance the precision of administration. Additionally, it eliminates the need for tail warming and restraint, thereby reducing the time required for administration.^11^ These advantages can significantly reduce the technical burden on researchers and potentially enhance the overall consistency and reliability of experimental results.

Future studies are warranted to validate the utility of retro‐orbital injection for establishing CKD models in other mouse strains. Furthermore, long‐term observations and more in‐depth histological analyses of renal functions will help elucidate the potential impact of different administration routes on CKD progression and long‐term prognosis.

In conclusion, retro‐orbital administration of 10 mg/kg ADR is recommended for establishing a CKD model in BALB/c mice due to its technical advantages and efficacy comparable to conventional tail‐vein administration. This technique is expected to improve experimental efficiency, reproducibility, and animal welfare in CKD research. As CKD research continues to advance, it is expected that this technique will contribute to more standardized and reliable experimental protocols, ultimately improving our understanding of the disease and the development of therapeutic strategies.

## AUTHOR CONTRIBUTIONS

**Masaki Watanabe:** Conceptualization; data curation; formal analysis; writing – original draft. **Hayato R. Takimoto:** Data curation; formal analysis. **Kazuki Hashimoto:** Data curation; formal analysis. **Yuki Ishii:** Data curation; formal analysis. **Nobuya Sasaki:** Conceptualization; project administration; supervision; writing – review and editing.

## FUNDING INFORMATION

This research received no external funding.

## CONFLICT OF INTEREST STATEMENT

All authors disclosed no conflict of interest that may directly or indirectly influence the content of the manuscript submitted.

## 5 ETHICS STATEMENT

The research was conducted in accordance with Kitasato University’s regulations for the care and use of laboratory animals. The president of Kitasato University approved the animal experiment protocol through the judgment of the Institutional Animal Care and Use Committee of Kitasato University (approval ID: 22‐059). Humane end points were established as follows: when mouse subjects exhibited moribundity or severe weight loss.
